# Supplementary material for: Ca2+ Homeostasis by Plasma Membrane Ca2+ ATPase (PMCA) 1 Is Essential for the Development of DP Thymocytes
Source: Int J Mol Sci. 2023 Jan 11;24(2):1442. doi: 10.3390/ijms24021442 (PMC9865543; doi:10.3390/ijms24021442)
Supplement: Supplementary file 1 [file ijms-24-01442-s001.zip › ijms-2101020-supplementary.pdf]

Supplementary Information

Ca^{2+} Homeostasis by Plasma Membrane Ca^{2+} ATPase (PMCA) 1 Is Essential for the Development of DP Thymocytes

David Beckmann [1], Kristina Langnaese [1], Anna Gottfried [1], Johannes Hradsky [1], Kerry Tedford [1], Nikhil Tiwari [2], Ulrich Thomas [2], Klaus-Dieter Fischer [1,*] and Mark Korthals [1]

[1] Institute for Biochemistry and Cell Biology, Medical Faculty, Otto-von-Guericke-University Magdeburg, 39120 Magdeburg, Germany
[2] Department of Cellular Neuroscience, Leibniz Institute for Neurobiology, 39120 Magdeburg, Germany
* Correspondence: klaus.fischer@med.ovgu.de

Supplementary Table S1: complete list of antibodies

**Primary antibodies**						
**Target**	**Clone**	**Host**	**Label***	**Application**	**Dilution**	**Company**
β-actin	AC-15	mouse	unlabeled	WB	1:10000	Sigma
active Caspase-3	polyclonal	rabbit	unlabeled	FC	1:100	R&D Systems
CD3ε	145-2C11	Armenian hamster	unlabeled/ LEAF	stimulation	10µg/ml	Biolegend
CD4	RM4-5	rat	Fluo	FC	1:200	Biolegend
CD5	53-7.3	rat	Fluo	FC	1:200	BD Biosciences
CD8	53-6.7	rat	Fluo	FC	1:200	Biolegend
CD11b	M1/70	rat	Fluo	FC	1:200	Biolegend
CD25	PC61	rat	Fluo	FC	1:200	Biolegend
CD44	IM7	rat	Fluo	FC	1:200	Biolegend
CD45R/B220	RA3-6B2	rat	Fluo	FC	1:200	Biolegend
CD62L	MEL-14	rat	Fluo	FC	1:200	Biolegend
CD69	H1.2F3	Armenian hamster	Fluo	FC	1:200	BD Biosciences
ERK-1/2	L34F12	mouse	unlabeled	WB	1:2000	Cell Signaling
FoxP3	FJK-16s	rat	Fluo	FC	1:50	Invitrogen/ eBioscience
Ki67	16A8	rat	Fluo	FC	1:200	Biolegend
Notch1	HMN1-12	Armenian hamster	Fluo	FC	1:200	Biolegend
Notch1 (IC)	mN1A	mouse	Fluo	FC	1:100	Biolegend
p53	REA609	recomb. human	Fluo	FC	1:50	Miltenyi Biotec
PMCA1	EPR12029	rabbit	unlabeled	IF<br>WB	1:100<br>1:5000	Abcam

PMCA4	JA9	mouse	unlabeled	WB	1:1000	Abcam
TCR β-chain	H57-597	Armenian hamster	Fluo	FC	1:200	Biolegend
TCR-yd	GL3	Armenian hamster	Fluo	FC	1:200	Biolegend
**Secondary antibodies**						
**Target**	**Clone**	**Host**	**Label**	**Application**	**Dilution**	**Company**
Rabbit IgG	polyclonal	donkey	Alexa Fluor 488	IF	1:500	Thermo Fisher
Hamster IgG	polyclonal $F(ab')_2$	goat	unlabeled	stimulation	variable	AbD Serotec
Mouse IgG	polyclonal	goat	POD	WB	1:10000	Jackson ImmunoResearch
Rabbit IgG	polyclonal	goat	POD	WB	1:10000	Jackson ImmunoResearch

FC, flow cytometry; FC Ca, flow cytometric ratiometric Ca^{2+} measurement; ICFC intracellular flow cytometry; IF, immunofluorescence; IP, immunoprecipitation; LEAF, low endotoxin, azid-free; POD, horseradish peroxidase; WB, Western blot. * Fluo, primary antibodies directly conjugated to one of the following fluorochromes: FITC, Alexa488, PE, PerCP, APC, V450, BV421, BV510, PE-Cy7, APC-Cy.

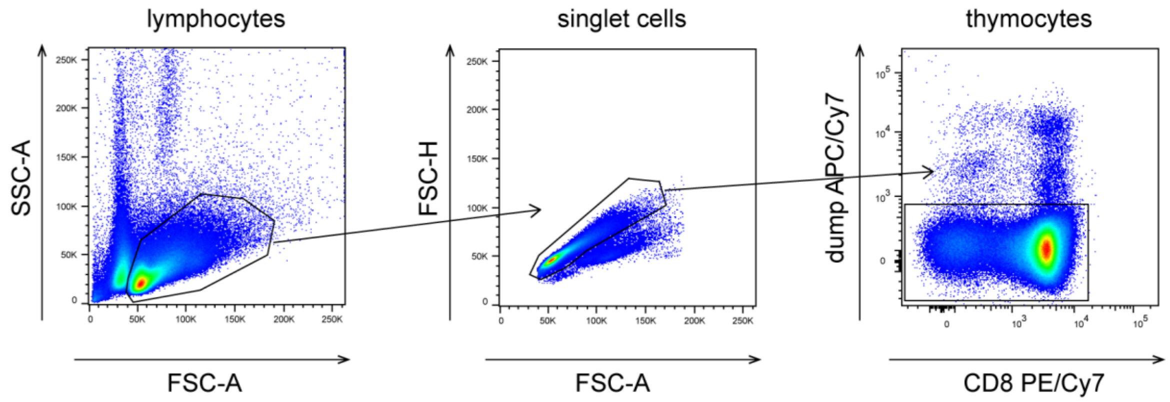

Supplementary Figure S1: Gating strategies. Lymphocytes obtained from thymi or secondary lymphoid organs were first gated by forward and side scatter (FSC-A/SSC-A) characteristics of viable leukocytes excluding most FSC^{low} dead cells and erythrocytes. Singlet cells were then gated according to FSC-H characteristics. Viable singlet thymocytes were finally gated by excluding B cells, myeloid cells, erythrocytes and dead cells using a dump antibody mix for B220, CD11b and Ter-119 and a dead cell labelling dye. The dump mix for Ca^{2+} measurements was varied in some experiments, e.g. by addition of CD4 and CD8 antibodies in order to also exclude DP and SP thymocytes and focus on DN thymocytes. Gating of subpopulations is shown in representative Plots in respective figures.

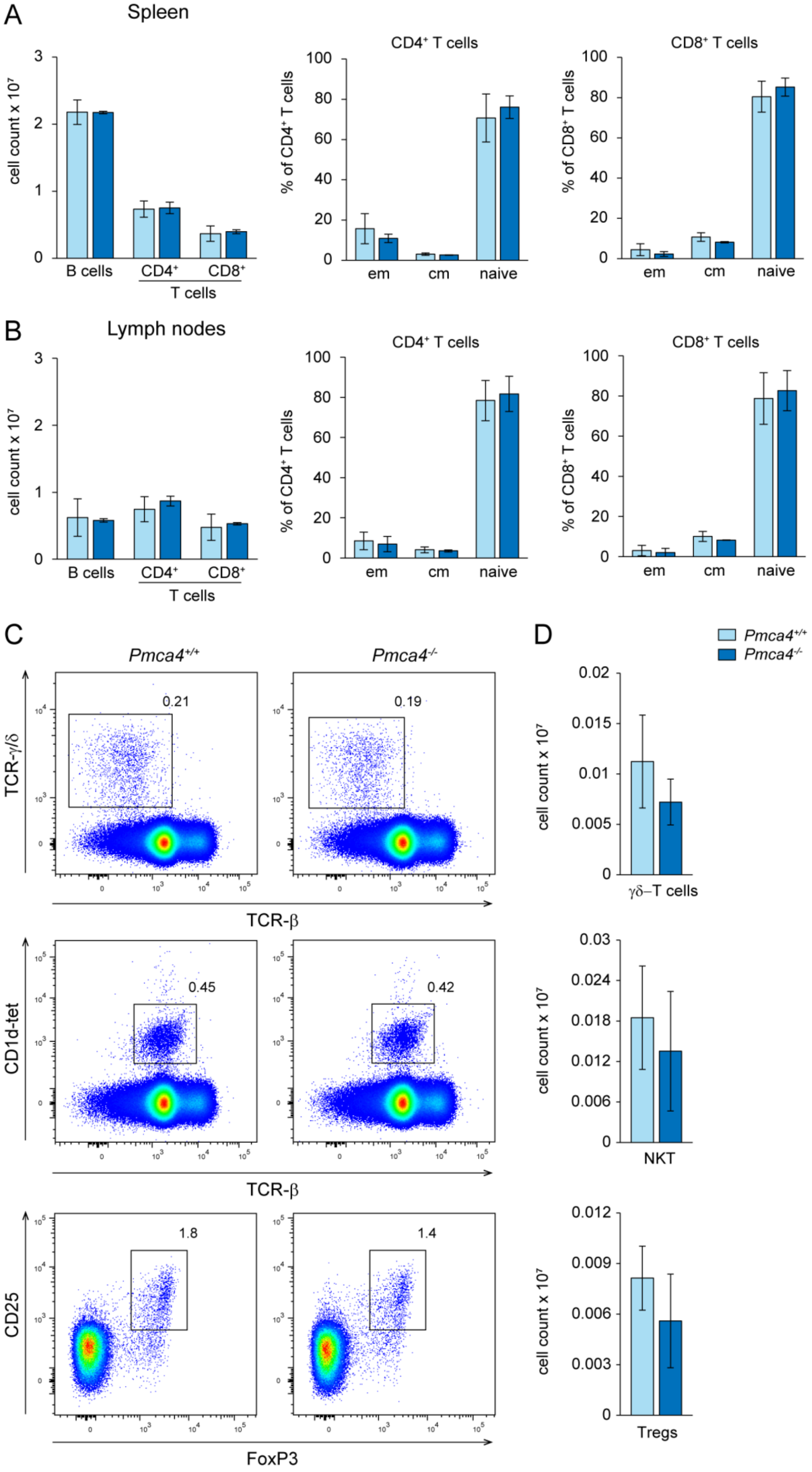

A
Spleen
cell count x 10⁷
B cells
CD4⁺
CD8⁺
T cells
CD4⁺ T cells
% of CD4⁺ T cells
em
cm
naive
CD8⁺ T cells
% of CD8⁺ T cells
B
Lymph nodes
C
Pmca4+/+
Pmca4-/-
TCR-γ/δ
0.21
0.19
TCR-β
CD1d-tet
0.45
0.42
CD25
1.8
1.4
FoxP3
D
γδ–T cells
NKT
Tregs

Supplementary Figure S2: Normal peripheral T cell numbers and normal development of unconventional T cells in the thymus of *Pmca4*$^{-/-}$ mice. **(A and B)** Bar graphs show total cell numbers of indicated cell populations in spleen (A) and lymph node (B) samples summarized as mean ± SD obtained from 2 experiments. Cells were gated as shown in Supplementary Figure 1 and 4, respectively. em, effector memory; cm, central memory. **(C and D)** FACS analysis of thymic γδ-T cells, NKT cells and Tregs. Representative FACS plots (C) showing thymocytes of *Pmca4*$^{+/+}$ and *Pmca4*$^{-/-}$ mice stained with anti-γδ-TCR antibody or PBS-57 loaded CD1d tetramer, or $CD4^+$ SP stained for CD25 and FoxP3 to label γδ-T cells (top row), NKT cells (middle row) or Tregs (bottom row), respectively. Numbers indicate the proportion of respective populations. Bar graphs (D) show mean total cell numbers ± SD of thymic γδ-T cells, NKT cells or Tregs in *Pmca4*$^{+/+}$ and *Pmca4*$^{-/-}$ mice obtained from 3 experiments.

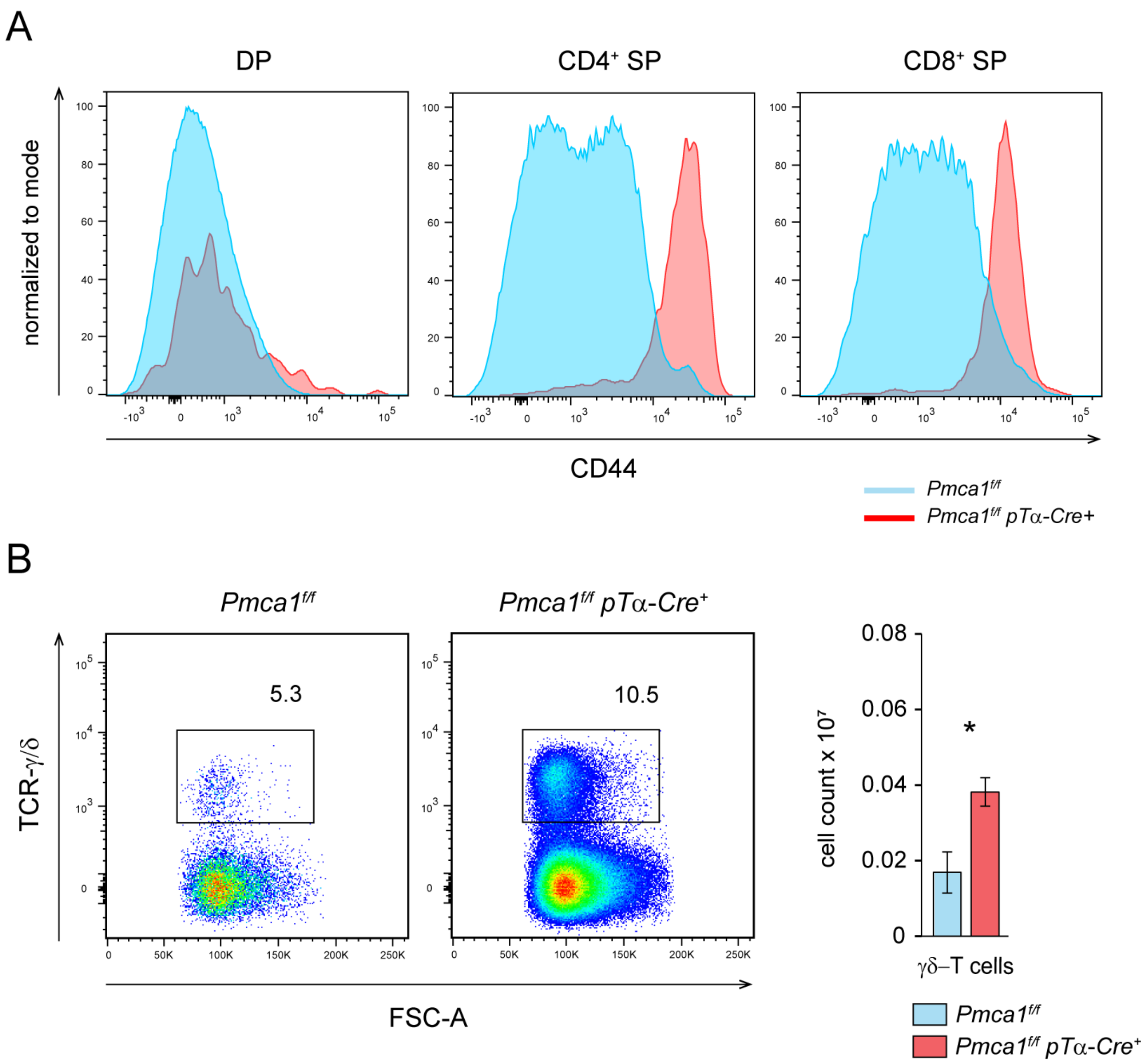

Supplementary Figure S3: Activated phenotype of SP thymocytes and favored γδ-T cell development in *Pmca1$^{f/f}$ pTα-Cre^{+}* mice. **(A)** Representative FACS histograms showing CD44 expression levels on DP and SP thymocytes of *Pmca1$^{f/f}$* and *Pmca1$^{f/f}$ pTα-Cre^{+}* mice. **(B)** FACS plots show TCR-γδ staining on DN thymocytes of *Pmca1$^{f/f}$* and *Pmca1$^{f/f}$ pTα-Cre^{+}* mice. The bar graph shows the mean ± SD total numbers of DN γδ-T cells quantified from 2 experiments. *$p<0.05$, by unpaired two-tailed t-test.

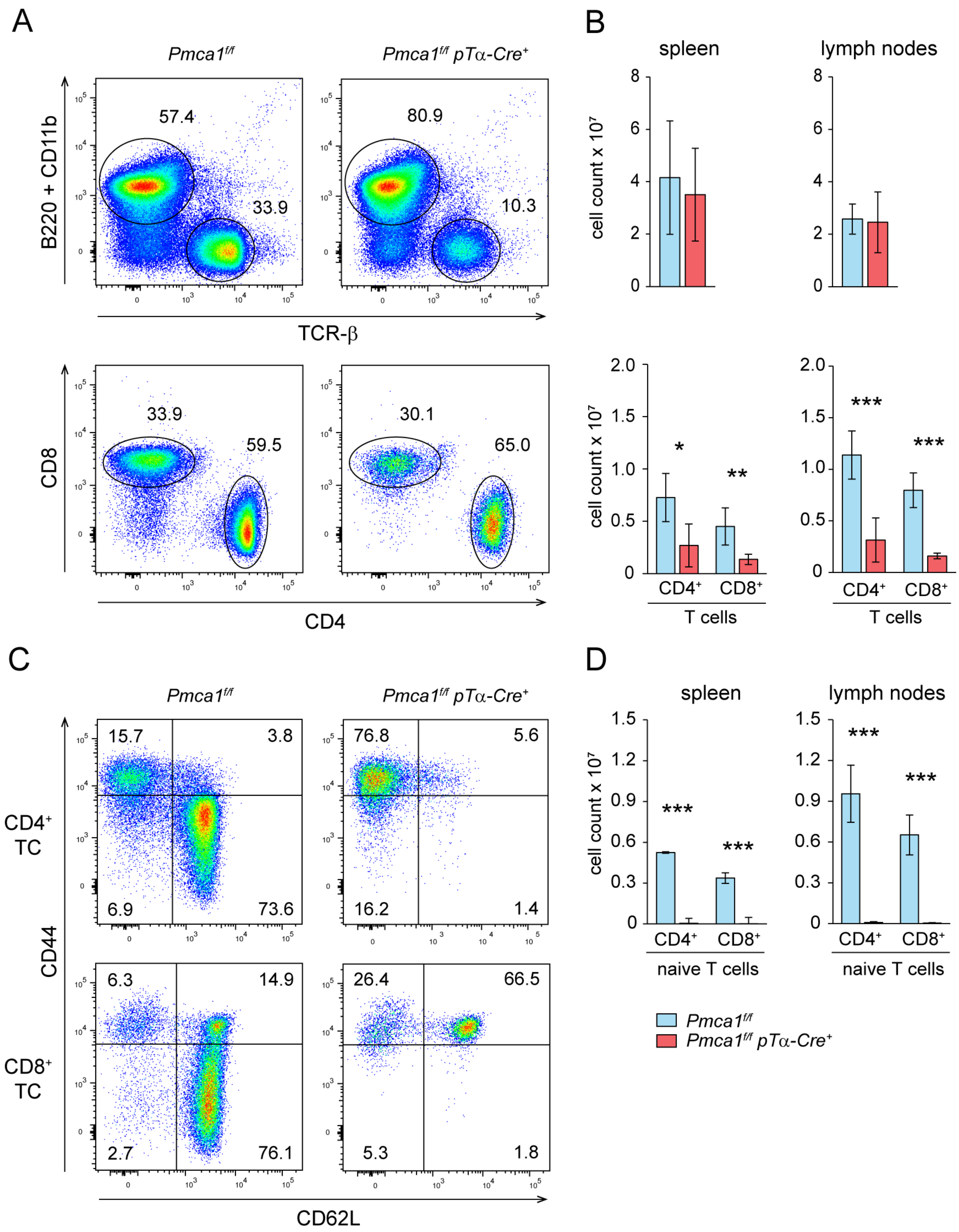

A
Pmca1f/f
Pmca1f/f pTα-Cre+
B220 + CD11b
57.4
33.9
80.9
10.3
TCR-β
CD8
33.9
59.5
30.1
65.0
CD4
B
spleen
lymph nodes
cell count x 10^7
CD4+
CD8+
T cells
C
Pmca1f/f
Pmca1f/f pTα-Cre+
CD4+ TC
CD8+ TC
CD44
15.7
3.8
6.9
73.6
76.8
5.6
16.2
1.4
6.3
14.9
2.7
76.1
26.4
66.5
5.3
1.8
CD62L
D
spleen
lymph nodes
cell count x 10^7
CD4+
CD8+
naive T cells
Pmca1f/f
Pmca1f/f pTα-Cre+

Supplementary Figure S4: *Pmca1*$^{f/f}$ *pTα-Cre*$^{+}$ mice lack naïve T cells. **(A)** Representative dot plots showing populations of TCR-β^{+} T cells and a combined population of B220^{+} B cells and CD11b^{+} myeloid cells (upper plots) and CD4^{+} versus CD8^{+} T cells (lower row) in splenocyte samples from *Pmca1*$^{f/f}$ and *Pmca1*$^{f/f}$ *pTα-Cre*$^{+}$ mice. Small numbers indicate frequencies of corresponding population within splenocytes or T cells, respectively. **(B)** Bar graphs summarize total cell numbers in spleen and lymph node samples obtained from 7 and 6 mice, respectively (upper row), and cell numbers of indicated T cell populations as mean ± SD obtained from 5 mice each (lower row). **(C)** Representative FACS plots of splenic CD4^{+} and CD8^{+} T cells stained for CD62L and CD44, labelling CD62L^{+}CD44^{low} naïve, CD62L^{-}CD44$^{+/high}$ effector memory and CD62L^{+}CD44^{high} central memory T cells of *Pmca1*$^{f/f}$ and *Pmca1*$^{f/f}$ *pTα-Cre*$^{+}$mice. **(D)** Bar graphs show mean total cell numbers ± SD of naïve CD4^{+} and CD8^{+} T cells in spleen and lymph node samples obtained from 5 mice each. *$p<0.05$, **$p<0.01$, ***$p<0.001$, all by unpaired two-tailed t-test.

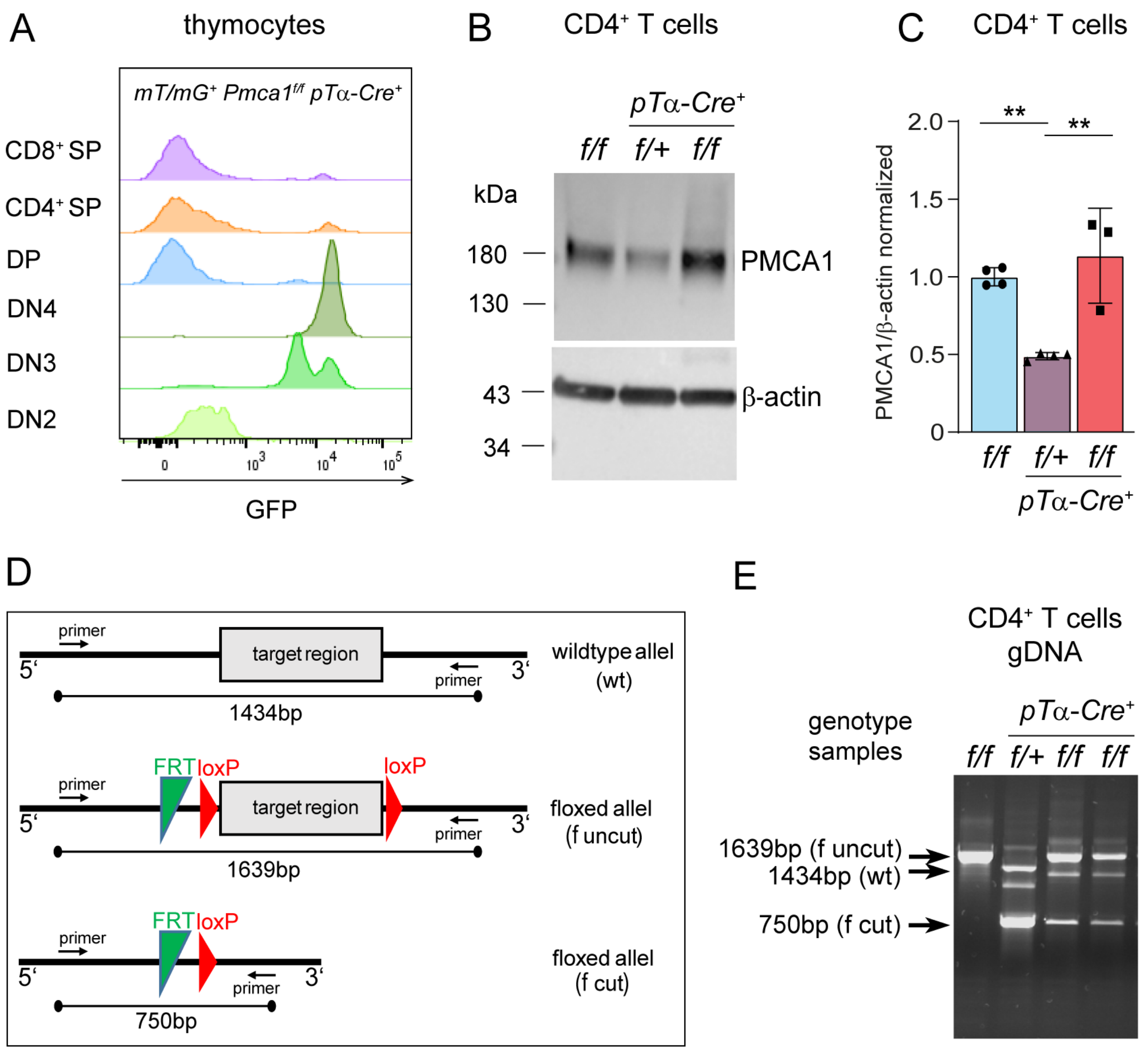

Supplementary Figure S5: Peripheral T cells in *Pmca1*$^{f/f}$ *pTα-Cre*$^{+}$ mice derive mainly from precursors that escaped *Pmca1* deletion. **(A)** Representative FACS histograms showing green GFP fluorescence of the mT/mG Cre reporter expressed in indicated thymocyte populations of *mT/mG*$^{+}$ *Pmca1*$^{f/f}$ *pTα-Cre*$^{+}$ mice. **(B and C)** Western analysis of PMCA1 protein expression in peripheral T cells. Purified splenic CD4^{+} T cells from *Pmca1*$^{f/f}$, heterozygous *Pmca1*$^{f/+}$ *pTα-Cre*$^{+}$ and homozygous *Pmca1*$^{f/f}$ *pTα-Cre*$^{+}$ mice were analyzed for PMCA1 expression and β-actin as loading control (B). OD of protein bands (n=4 for *f/f*, n=4 for *f/+ pTα-Cre*$^{+}$ and n=3 for *f/f pTα-Cre*$^{+}$) was quantified and divided by the intensity of the corresponding β-actin band. Bar graphs (C) show mean relative protein amounts ± SD normalized to the mean obtained from control cells. **P <

0.01, by ANOVA. **(D and E)** Analysis of pTα-Cre activity in target $CD4^+$ T cells by genomic PCR. The diagram in (D) illustrates sizes of the expected PCR products from genomic DNA for the wildtype allele as well as for the floxed allele either uncut or Cre cut, respectively. Genomic DNA of $CD4^+$ T cells obtained from mice with indicated genotypes was analyzed using this PCR strategy (E), demonstrating inefficient Cre activity in target $CD4^+$ T cells from two *Pmca1*$^{f/f}$ *pTα-Cre*$^+$ mice.

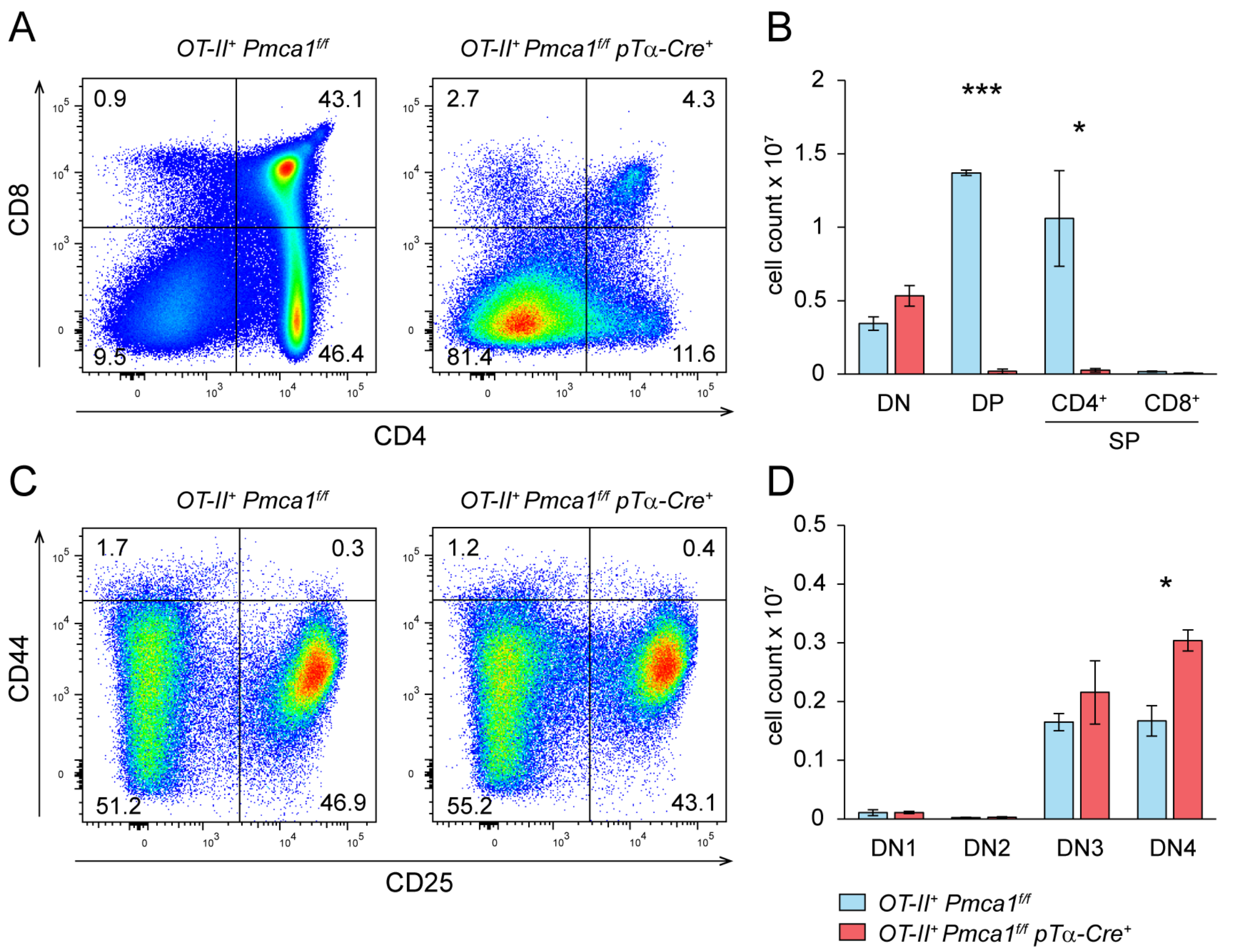

Supplementary Figure S6: Transgenic expression of a recombinant TCR does not rescue the developmental block in thymocytes of *Pmca1$^{f/f}$ pTα-Cre^{+}* mice. **(A)** Representative FACS plots of CD4 and CD8 stained thymocytes and **(B)** quantification of total numbers of DN, DP and CD4^{+} and CD8^{+} SP thymocytes obtained from thymi of *OT-II^{+} Pmca1$^{f/f}$* or *OT-II^{+} Pmca1$^{f/f}$ pTα-Cre^{+}* mice. **(C)** Representative CD44/CD25 FACS plots showing DN1 to DN4 thymocyte populations and **(D)** quantification of total numbers of these populations in thymi of *OT-II^{+}*-transgenic *Pmca1$^{f/f}$* or *Pmca1$^{f/f}$ pTα-Cre^{+}* mice. All diagrams show mean absolute cell numbers ± SD of indicated thymocyte populations combined from 2 experiments. *$p<0.05$, ***$p<0.001$, all by unpaired two-tailed t-test.

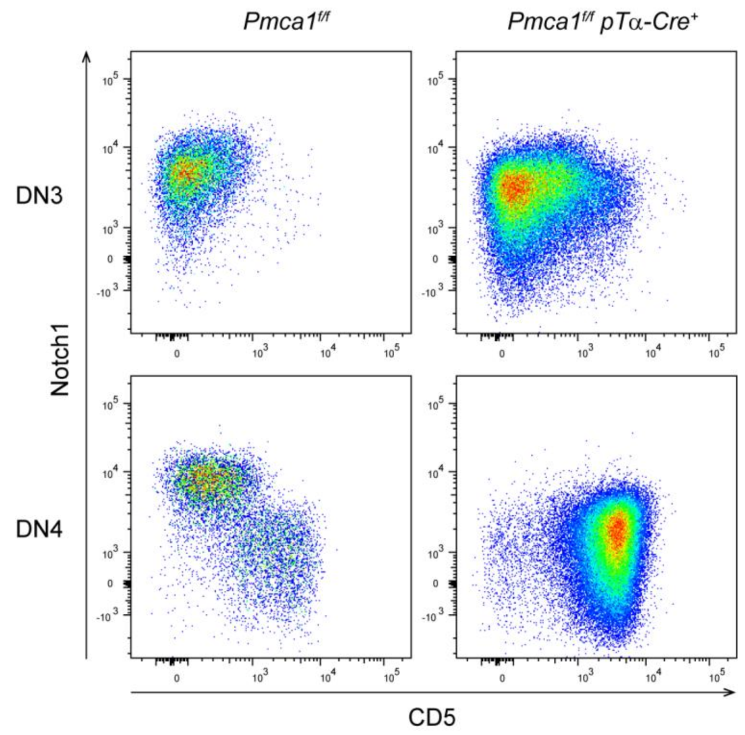

Supplementary Figure S7: Inverse correlation of Notch1 and CD5 expression in wildtype DN4 thymocytes. Representative FACS dot plots show Notch1 and CD5 staining of DN3 and DN4 thymocytes from *Pmca1$^{f/f}$* or *Pmca1$^{f/f}$ pTα-Cre^{+}* mice.

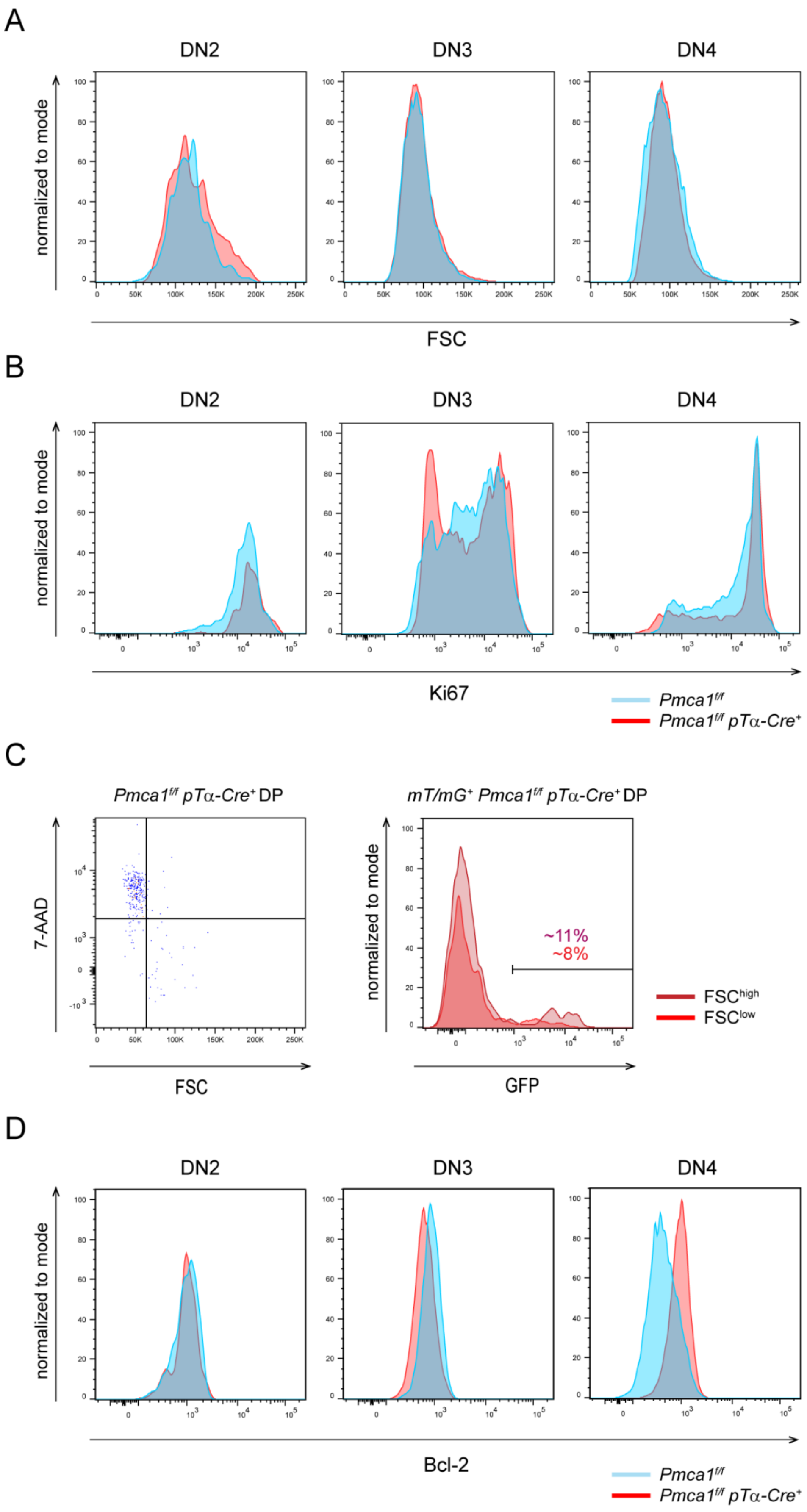
A
DN2
DN3
DN4
normalized to mode
FSC
B
DN2
DN3
DN4
normalized to mode
Ki67
Pmca1f/f
Pmca1f/f pTα-Cre+
C
Pmca1f/f pTα-Cre+ DP
mT/mG+ Pmca1f/f pTα-Cre+ DP
7-AAD
FSC
normalized to mode
~11%
~8%
GFP
FSChigh
FSClow
D
DN2
DN3
DN4
normalized to mode
Bcl-2
Pmca1f/f
Pmca1f/f pTα-Cre+

Supplementary Figure S8: Unaltered proliferative capacity and persistent survival of DN thymocytes but apoptosis of both PMCA1 deficient and PMCA1 expressing DP thymocytes in *Pmca1*$^{f/f}$ *pTα-Cre*$^{+}$ mice. **(A)** Representative FACS histograms showing FSC intensities of indicated thymocyte populations of *Pmca1*$^{f/f}$ or *Pmca1*$^{f/f}$ *pTα-Cre*$^{+}$ mice. **(B)** Representative FACS histograms showing intracellular Ki67 staining patterns in indicated thymocyte populations of *Pmca1*$^{f/f}$ or *Pmca1*$^{f/f}$ *pTα-Cre*$^{+}$ mice. **(C)** Extremely rare actual PMCA1 deficient dead DP thymocytes were identified using low FSC as a surrogate marker for dead cells and GFP expression in *Pmca1*$^{f/f}$ *pTα-Cre*$^{+}$ mice also expressing the Cre-reporter. The FACS plot (left) shows good correlation of 7-AAD staining and low FSC in total DP thymocytes of *Pmca1*$^{f/f}$ *pTα-Cre*$^{+}$ mice. The FACS histogram (right) shows GFP expression within the FSC^{high} viable and the FSC^{low} dead DP thymocyte population of *mT/mG*$^{+}$ *Pmca1*$^{f/f}$ *pTα-Cre*$^{+}$ mice. **(D)** Representative FACS histograms showing intracellular Bcl-2 fluorescence intensities in indicated thymocyte populations of *Pmca1*$^{f/f}$ or *Pmca1*$^{f/f}$ *pTα-Cre*$^{+}$ mice.
